# Supplementary material for: A Critical View of the Application of the APEX Software (Aqueous Photochemistry of Environmentally-Occurring Xenobiotics) to Predict Photoreaction Kinetics in Surface Freshwaters
Source: Molecules. 2019 Dec 18;25(1):9. doi: 10.3390/molecules25010009 (PMC7017383; doi:10.3390/molecules25010009)
Supplement: Supplementary file 1 [file molecules-25-00009-s001.zip › 3.2.4_gcc-4.4.0.zip!/3.2.4_gcc-4.4.0/3.2.4_gcc-4.4.0/doc/octave/html/interpreter/Example-Codes.html]

Example Codes - Untitled


Next: Zeros Treatment,
Previous: Function Support,
Up: Diagonal and Permutation Matrices


---

### 20.4 Some Examples of Usage

The following can be used to solve a linear system `A*x = b`
using the pivoted LU factorization:

```
       [L, U, P] = lu (A); ## now L*U = P*A
       x = U \ L \ P*b;
```

This is how you normalize columns of a matrix X to unit norm:

```
       s = norm (X, "columns");
       X = diag (s) \ X;
```

The following expression is a way to efficiently calculate the sign of a
permutation, given by a permutation vector p. It will also work
in earlier versions of Octave, but slowly.

```
       det (eye (length (p))(p, :))
```

Finally, here's how you solve a linear system `A*x = b`
with Tikhonov regularization (ridge regression) using SVD (a skeleton only):

```
       m = rows (A); n = columns (A);
       [U, S, V] = svd (A);
       ## determine the regularization factor alpha
       ## alpha = ...
       ## transform to orthogonal basis
       b = U'*b;
       ## Use the standard formula, replacing A with S.
       ## S is diagonal, so the following will be very fast and accurate.
       x = (S'*S + alpha^2 * eye (n)) \ (S' * b);
       ## transform to solution basis
       x = V*x;
```
